# Human rights of persons with mental illness in Indonesia: more than legislation is needed

**DOI:** 10.1186/1752-4458-3-14

**Published:** 2009-06-19

**Authors:** I Irmansyah, YA Prasetyo, H Minas

**Affiliations:** 1 Department of Psychiatry, University of Indonesia, Kimia II No 35, Jakarta, 10430, Indonesia; 2 National Human Rights Commission, Jalan Latuharhari 4B, Menteng, Jakarta Pusat 10310, Indonesia; 3 Centre for International Mental Health, Melbourne School of Population Health, University of Melbourne, Parkville, Victoria 3010, Australia; 4 National Taskforce on Mental Health System Development in Indonesia, Indonesia

## Abstract

**Background:**

Although attention to human rights in Indonesia has been improving over the past decade, the human rights situation of persons with mental disorders is still far from satisfactory. The purpose of this paper is to examine the legal framework for protection of human rights of persons with mental disorder and the extent to which Indonesia's international obligations concerning the right to health are being met.

**Methods:**

We examined the Indonesian constitution, Indonesian laws relevant to the right to health, the structure and operation of the National Human Rights Commission, and what is known about violations of the human rights of persons with mental illness from research and the media.

**Results:**

The focus of the Indonesian Constitution on rights pre-dated the Universal Declaration, Indonesia has ratified relevant international covenants and domestic law provides an adequate legal framework for human rights protections. However, human rights abuses persist, are widespread, and go essentially unremarked and unchallenged. The National Human Rights Commission has only recently become engaged in the issue of protection of the rights of persons with mental illness.

**Conclusion:**

More than legislation is needed to protect the human rights of persons with mental illness. Improving the human rights situation for persons with mental illness in Indonesia will require action by governments at national, provincial and district levels, substantial increases in the level of investment in mental health services, coordinated action by mental health professionals and consumer and carer organisations, and a central role for the National Human Rights Commission in protecting the rights of persons with mental illness.

## Background

For many years Indonesia has been subject to international scrutiny because of human rights violations. During the political turmoil in 1965, which brought Soeharto to power and 34 years as president, many hundreds of thousands of people were killed, and the consequences for survivors and their children persist more than 40 years later. During the Soeharto era a number of political movements emerged, such as Tj Priok movement, Talangsari, Lampung, East Timor [1] and GAMH in Aceh. The usual response from government was military action resulting in great suffering and loss of life.

After the fall of the New Order regime the political transformation of Indonesia into a competitive, multi-party system has led to major changes in state institutions and in the relationships between the state and civil society. However, "the public sector continues to be characterized by inefficiencies, corruption, and lack of regard for the needs of the society at large" [2]. There is a need for continuing and substantial structural and procedural reforms in the systems of public administration.

Since the establishment of the National Commission on Human Rights [3] in 1993 attention to human rights in Indonesia has improved significantly. The constitution has been amended to accomodate the key principles of the Universal Declaration on Human Rights. In 1999 Law #39 on Human Rights was enacted. Indonesia has ratified the International Convenant on Civil and Political Rights (ICCPR) [4] and the International Convenant on Social, Economic and Cultural Rights (ICSECR) [5]. In 2006 Indonesia became a member of UN Human Rights Council and the UN Security Council, emphasising Indonesia's formal commitment to human rights.

### The situation of people with mental illness in Indonesia

Unfortunately the situation of persons with mental disorders in Indonesia is still far from satisfactory from a human rights perspective. Even basic mental health services are not available in many parts of the country. Many people with mental illness have no access to treatment. Primary health services do not have mental health as a priority and the skills of primary health clinicians are not sufficient to ensure detection and appropriate treatment of mental disorder. Some persons with mental illness are confined and restrained in the community [6] in inhumane ways. The quality of mental health services in hospitals is generally poor and human rights protections for patients are weak. Custodial treatments dominate in psychiatric hospitals. Involuntary treatment is common, even though there is no legal basis for involuntary admission. A person can be brought to hospital without his or her consent by anybody who feels uneasy about the person's behaviour. There are no guardianship laws or arrangements and there is no requirement for legal review of the need for involuntary hospitalisation and treatment. As in many developing countries, standards of care are poor and, failure to protect the basic human rights of people with mental illness is common.

## Methods

In order to explore the human rights situation of people with mental illness in Indonesia we commenced with an examination of the Indonesian Constitution and the amendments to the Constitution, reviewed relevant international law which has been ratified by Indonesia, examined the composition and functions of the National Human Rights Commission and its annual reports, and reviewed relevant laws and regulations which have been enacted by national or provincial goverments. We searched relevant research conducted in Indonesia. Most of the research has not been published in international journals, and is to be found in local academic institutions as theses or in reports of national scientific meetings. Most of the research reports are in the national language, Bahasa Indonesia, and inaccessible to foreigns scholars who are not Bahasa speakers. We also explored local media reports on human rights violations experienced by people with mental illness.

## Results

### The Indonesian constitution

The 1945 Constitution of the Republic of Indonesia, [7] called Undang Undang Dasar 45, named after the year of Indonesian independence, was a very short and simple document consisting of 37 articles, of which six explicitly dealt with human rights (Articles 26–31) (Tables 1). It is worth noting that the commitments to human rights enunciated by the constitution pre-dated the 1948 United Nations Universal Declaration of Human Rights. The rights mentioned were to be established by law, and could also be subject to restriction by law [8]. A prominent example of such restriction has been the previous restriction by law of freedom of speech in the press, contrary to Article 28.

**Table 1:** Human rights protections in the 1945 Constitution

**Relevant chapters from the Constitution**	
CHAPTER X: CITIZENSHIP	**Article 27**
	(1). All citizens, without exception, shall be equal before the law and in government and shall have the duty to respect the law and the government
	(2). Every citizen shall have the right of employment and to a living befitting human beings.
	**Article 28**
	Freedom of association and assembly, of expressing thoughts by speech and writing, and so on, shall be laid down by law.

CHAPTER XI: RELIGION	**Article 29**
	(2). The state shall guarantee freedom to every resident to adhere to their respective religion and to perform their religious duties in accordance with their religion and that faith.

CHAPTER XII: DEFENSE	**Article 30**
	(1). Every citizen shall have the right and the duty to participate in the defense efforts of the state.

CHAPTER XIII: EDUCATION	**Article 31**
	(1). Every citizen shall have the right to an education.
	(2). The government shall establish and operate a national education system which shall be provided for by law.

The constitution has been amended four times since the commencement of the reformation era (1998). Presidential Decree No. 129 of 1998 concerning the National Human Rights Plan, dealt with preparation for the ratification of international human rights instruments; dissemination of information and education on human rights; implementation of priority issues on human rights, and implementation of the international human rights instruments which had been ratified by Indonesia [9].

In 2002 human rights articles were inserted in the constitution in the second amendment and there is now a specific chapter on human rights (Chapter XA) (Table 2). The articles in this chapter incorporate almost all the basic human rights principles from the Universal Declaration on Human rights [8,10]. The amendment signalled an enhanced commitment by the country to the protection of the human rights of Indonesian citizens.

**Table 2:** Second Amendment to the 1945 Indonesian Constitution

**CHAPTER XA: HUMAN RIGHTS**	
Article 28A	Every person shall have the right to live and to defend his/her life and livelihood.

Article 28B	(1) Every person shall have the right to build a family and to have descendant through a legal marriage.
	(2) Every child shall have the right to live, to grow, and to be protected against violence and discrimination.

Article 28C	(1) Every person shall have the right to improve himself/herself through fulfilment of basic needs, and entitled to an education and to obtain benefit from science and technology, art and culture, in order to enhance his/her quality of life, for the sake of human welfare.
	(2) Every person shall have the right to advance himself/herself by defending his/her rights collectively and develop his/her society, nation, and country.

Article 28D	(1) Every person shall have the right for recognition, guarantees, protection, and a just legal certainty as well as equal treatment before the law.
	(2) Every person shall have the right of employment opportunities and receive a just and reasonable compensation from the employment relationship.
	(3) Every citizen shall have the right to obtain equal opportunities in governance.
	(4) Every person shall have the right of citizenship status.

Article 28E	(1) Every person shall be free to adhere to his/her respective religion and perform worship according to his/her religion, choose his/her education and learning, choose his/her work, choose citizenship, choose to reside within the nation's territory and depart from it, and is entitled to return.
	(2) Every person shall have the right to have freedom of belief, express his/her thoughts and attitudes, in accordance with his/her conscience.
	(3) Every person shall have the right of freedom to organize, to assemble, and to express opinions.

Article 28F	Every person shall have the right to communicate and to obtain information to develop his/her personality and social environment, as well as the right to seek, to obtain, to possess, to keep, to process, and to convey information by utilizing all available kinds of channels.

Article 28G	(1) Every person shall have the right of self-protection, family, honor, dignity, and property under his/her authority, as well as entitled to a feeling of safety and protection from threats of fear to do or not to do anything according to the basic rights.
	(2) Every person shall have the right to be free from torture or any derogatory treatment demeaning human dignity and is entitled to political asylum from another nation.

Article 28H	(1) Every person shall have the right to live in welfare both physically and spiritually, have a place to reside, and receive a proper and healthy environment, as well as receive medical care.
	(2) Every person shall have the right of facilities and special treatment for equal opportunities and benefits in order to achieve equality and equity.
	(3) Every person shall have the right of social security guarantees that enable him/her to develop completely as a dignity human being.
	(4) Every person shall have the right of personal possessions and those possessions shall not be confiscated arbitrary by any person whatsoever.

Article 28I	(1) The right for living, the right for not being tortured, the right for freedom of thought and conscience, religious rights, the right for not being enslaved, the right for being recognized as an individual before the law, and the right for not being prosecuted based on retroactive laws shall be the rights as human that may not be diminished in any situation whatsoever.
	(2) Every person shall have the right to be free from discriminatory treatment on the basis of any pretext and is entitled to receive protection from that discriminatory treatment.
	(3) The cultural identity and traditional society rights shall be respected in line with age progress and human civilization.
	(4) The protection, advancement, upholding, and fulfilment of human rights shall be the responsibility of the state, especially the government.
	(5) To uphold and to protect human rights in accordance with the principles of a legal democratic nation, the practice of human rights shall be guaranteed, arranged, and embodied in statutory laws.

Article 28J	(1) Every person shall have the duty to respect the others' human rights within the orderly context of living in a community, nation, and state.
	(2) In carrying out rights and freedoms, every person is required to obey the predetermined limitations regulated by the law for the sole purpose of guaranteeing recognition and respect over the rights and freedoms enjoyed by other people and to fulfil the just demands in accordance with the considerations of morals, security, and public order within a democratic society.

### National Commission on Human Rights and Human Rights Law

A structure for ensuring attention to human rights in Indonesia was created with the establishment in 1993 of the National Human Rights Commmission (Komnas HAM) [11]. The Commission was established by Presidential Decree No. 50 in 1993, with members of the Commission directly appointed by the president. The Commission was strengthened in a number of ways following passage of the Human Rights Act (Law no. 39 of 1999).

The broad functions of the Commission are:

(a) to develop conditions conducive to the execution of human rights in accordance with Pancasila, the 1945 Constitution, the United Nations Charter, and the Universal Declaration of Human Rights; and

(b) to improve the protection and upholding of human rights in the interests of the personal development of Indonesian people as a whole and their ability to participate in several aspects. In order to achieve these aims, "the National Commission functions to study, research, disseminate, monitor and mediate human rights issues" (Art. 76(1).

The specific objectives of the Commission, as specified in the Act (article 89) are to:

(a) study and examine international human rights instruments with the aim of providing recommendations concerning their possible accession and ratification;

(b) study and examine legislation in order to provide recommendations concerning drawing up, amending and revoking of legislation concerning human rights;

(c) publish study and examination reports;

(d) carry out literature studies, field studies, and comparative studies with other countries;

(e) discuss issues related to protecting, upholding and promoting human rights; and,

(f) conduct cooperative research and examination into human rights with organizations, institutions and parties, at regional, national and international levels.

As part of its monitoring functions the Commission can investigate and examine incidents likely to constitute violations of human rights; call on complainants, witnesses and accused to hear their statements and to give written statements and submit necessary documents; and survey places of incidents. The Commission receives complaints and can decide to set up *ad hoc *teams to look at cases of gross and widespread human rights violations.

In 2006 Indonesia ratified the International Covenant on Civil and Political Rights (ICCPR) [4] and the International Convenant on Social, Economic and Cultural Rights (ICSECR) [5]. Almost at the same time Indonesia became a full member of UN Human Rights Council and UN Security Council. Indonesia ratified the Conventon on the Rights of People with Disability in 2007. Other relevant international conventions that have been ratified by Indonesia are: the UN Convention Against Tortune (CAT) [12], 1998, and the Convention on the Elimination of Racial Discrimination (1999) [13]. The process for ratification of the UN Optional Protocol to the Convention Against Torture and other Cruel, Inhuman or Degrading Treatment or Punishment (2002) is in progress. Ratification of these international instruments incorporates their provisions into Indonesian law, with all of the obligations that this implies.

However, ratification of international human rights instruments does not equate with implementation and real protection. "Indonesia's justice institutions – the police, prosecution and judiciary – are all in a state of collapse, incapable of upholding the law and protecting citizens' rights... Indonesia has much work to do before its citizens' rights under the ICCPR and ICESCR can be realised" [14].

### Human rights and health legislation

The awareness of health as a basic human right, within the population in general and within political parties, has increased over time. Law #23 on Health was passed in 1992. The main aim of this legislation was to bring into one act all regulations on health which were previously scattered across different pieces of legislation. Specific mental health law, enacted in 1966, well before many other countries in the region, was repealed. The 1966 mental health act included provisions for protection of the rights of persons with mental disorders, including the right to adequate treatment and rehabilitation, and was explicit concerning the obligation of the state to support mental health promotion and illness prevention. A consequence of this change was that legislative provisions specific to mental health were greatly reduced, to just four articles in the health law (Law #23 of 1992) Table 3).

**Table 3:** Mental health provisions in the general health law (Law #23) of 1992 (Chapter 7)

Chapter 7, Law #23 (Health)	
Article 24	1. Mental health are granted to achieve optimal, intelectual and emotional mental health state
	2. Mental health activities include maintaining and improving mental health, prevention and management of psychosocial problems and mental disorders, treatment and rehabiitation of mental disorders.
	3. Activities on Mental health are carried out by individual, family, school, work place, community members, and are supported by mental health services and other facilities.

Article 25	1. Government provides treatment and hospitalization, and gives support to the person who has recovered to return to the community.
	2. Government encourages, supports and supervises community activities on the prevention and intervention of psychosocial problems and mental disorders, and recovery process of the person with mental disorder to return to the comunity

Article 26	1. Persons with mental disorder who are considered dangerous and disturb the community have to be treated and hospitalized in the mental health service facilities or in other health services.
	2. Treatment and hospitalization of person with mental health problems could be requested by husband or wife or the guardian or other family members or by person who is responsible for local security, or by court if the detainee has mental disorder.

Article 27	1. Goverment will provide a presidential decree for other regulations and the management of mental health.

The expected benefit was that coordination of health policy would be improved and mental health would more clearly and explicitly become part of the general health system. Unfortunately this expected benefit has not been realised. One of the reason could be that the articles are too general and difficult to apply. For example, the first two articles (article #24 and #25) are general principles on mental health that need to be translated into regulations to implement them.

On the other hand, Article #26 could disadvantage persons with mental illness since there is no further explanation how to protect the right of information and consent for persons with mental health problems. Anybody can take a person with mental health problems to a mental hospital without provision of mechanisms for review of decisions to admit or duration of stay. This could contribute to explaining the observation that prolonged hospitalisation is common in psychiatric institutions in Indonesia. Article #26 also creates the strong impression that persons with mental illness are dangerous and need to be isolated in a mental hospital.

There is no clear definition of mental disorders or guidance on determining whether a person has a mental disorder.

Article #27 of the 1992 health law specifies that "Government will provide a presidential decree for other regulations and the management of mental health". However, after more than 17 years after this law was enacted no such decree has been issued.

Coverage of mental health issues in the health law is insufficient. Some provisions considered by the World Health Organisation to be essential in mental health legislation, and which are not part of Indonesian health law, include: [13]

• Protection of the rights of persons with mental disorders.

• The principle of least restrictive treatment

• Informed consent and confidentiality

• Independent review mechanisms

• Issues of community-based services

• The role of families and other carers

• The role of other sectors in the promotion of mental health and the prevention of mental disorders such as housing, education, employment and general health.

The health legislation is already outdated and needs amendment. Intensive discussions on amending the health act started in 2000. Significant progress has been made, including suggested improvements in the mental health chapter. Some mental health professionals, members of the Mental Health Advocacy Network, have consistently proposed a more comprehensive mental health chapter and have submitted proposals for amendment in the relevant form (academic paper) to the legislature [15]. Up to this point the proposed amendments have not been adopted.

There are several other pieces of legislation that are specifically relevant to health and mental health.

Law #29, 2004, on Medical Practice has the objective of protecting patients and service providers and improving the quality of health services. For many years, doctors dominated the doctor-patient relationship and sometimes exercised absolute power in decision making. During the last decade patients have become more assertive and now commonly expect an equal position in the doctor-patient relationship. Some patients have instituted legal proceedings for malpractice. The law on medical practice is potentially an important instrument for protection of persons with mental health problem from human rights abuses during treatment. The Law on Drug Abuse (Law #22, 1997), Law on Psychotropics (Law #5, 1997), Law on Child Protection (Law #23, 2002), and Law on Domestic Violence (Law #23, 2004) are all potentially relevant to mental health. Other legislation relevant to mental health is currently under discussion, including legislation on hospitals, professionals, and on drugs.

Unfortunately there are some laws that explicitly violate the human rights of people with mental disorder. Article #14(2a) of the legislation that governed general elections (Law #12, 2003) removed the right to vote from people with mental disorder. Fortunately, this article has been removed from the new legislation on general elections (Law #10, 2008). Another example of discriminatory legislation is the law on public regulations at the Jakarta provincial level which states that people with mental illness are forbidden from being in public places (article 41, Perda Tibum, Law #8, 2003) [15]. This legislation is currently being revised.

Although there is now no specific mental health legislation the provisions of other laws and regulations in Indonesia can serve to protect the human rights to people which mental illness.

### Violation of human rights of persons with mental illness

In November 2003 TIME Asia published a special report on the terrible situation of mental health in Asia, including in Indonesia [16]. The article and the accompanying photoessay [17] depicted patients in an Indonesian institution for people with mental illness who were chained and were being held in clearly unacceptable conditions.

The institution featured in the Time Asia story and photoessay, managed by the Jakarta Social Agency to house and care for people with chronic mental illness, and other similar institutions in Jakarta and East Java, was again the subject of attention by the Jakarta media in 23–24 May 2009 [18] when it was revealed in a report published by the Jakarta's Social Agency that large numbers of residents had been dying as a result of over-crowding, diarrhoea and malnutrition.

People with mental illness are also restrained and confined in the community [6], a practice known as *pasung*, in which people with mental illness are chained, tied, confined in small rooms or sheds, or have their legs in wooden stocks [6,19]. In the province of Aceh there are currently 110 known cases of *pasung *[20] although the actual number is probably substantially greater.

Access to health services is a the basic human right. In Indonesia, as in many other countries, access to mental health services is far from adequate for most of the population. A study by Heriani et al showed the long duration of untreated psychosis and a complex pathway to treatment of person with schizophrenia as a product of lack of access to mental health sevice [21] due to the lack of mental health programs in primary health centers and the lack of other community based services.

Other examples of violation of the rights of people with mental illness include insufficient and poor quality treatment and care in mental hospitals, the use of unmodified ECT, isolation and sometimes restraint of patients in hospital beds, the routine locking of hospital wards at 5 pm. A survey by Darmono et al [22] showed that people with mental illness experienced aggressive and violent behaviour even by hospital staff.

These conditions and practices clearly violate article 7 of ICCPR [4] (*No one shall be subjected to torture or to cruel, inhuman or degrading treatment or punishment. In particular, no one shall be subjected without his free consent to medical or scientific experimentation.) *and article 12 of IESCR [5]* (1. The States Parties to the present Covenant recognise the right of everyone to the enjoyment of the highest attainable standard of physical and mental health. 2. The steps to be taken by the States Parties to the present Covenant to achieve the full realisation of this right shall include those necessary for... d) The creation of conditions which would assure to all medical service and medical attention in the event of sickness.)*.

An examination of reports made to the National Human Rights Commission demonstrates the general lack of attention to the rights of people with mental illness. In 2006 there were 1,451 of reports submited to the Commission [23]. Only two of these reports concerned health issues and none was concerned with mental health issues. In 2007, there were no reports that related to the rights of people with mental illness [24].

### State obligations

The Right to Health was affirmed at the international level in Article 25 of the Universal Declaration of Human Rights in 1948. The United Nations expanded upon the "Right to Health" in Article 12 of the International Covenant on Economic, Social and Cultural Rights in 1966. Article 12 of the Covenant recognises the right of everyone to "the enjoyment of the highest attainable standard of physical and mental health." Article 12.2 requires States parties to take specific steps to improve the health of their citizens. The Committee on Economic, Social and Cultural Rights has, in General Comment 14 [25], extensively elaborated on the obligations of States parties to implement Article 12 of ICESCR [5]. The Committee emphasises that the entitlements under Article 12 "include the right to a system of health protection which provides equality of opportunity for people to enjoy the highest attainable level of health."

Examples of the legal obligations of governments that are identified in General Comment 14 [25] include, for example, rights to adequate facilities, equipment and supplies (e.g. drugs) and skilled staff, appropriate mental health treatment and care, non-discrimination and equal treatment, gender equality and the rights of children and adolescents, and the rights of persons with disabilities. While it is recognised that progressive realisation of these obligations is inevitable, particularly due to limits in resources, Article 12 imposes on States parties various obligations which are of immediate effect. "States parties have immediate obligations in relation to the right to health, such as the guarantee that the right will be exercised without discrimination of any kind and the obligation to take steps towards the full realization of article 12. Such steps must be deliberate, concrete and targeted towards the full realization of the right to health... States parties have a specific and continuing obligation to move as expeditiously and effectively as possible towards the full realization of article 12." National, provincial and district governments in Indonesia could be more expeditious and effective in realising their obligations under Article 12 of ICESCR.

"The right to health, like all human rights, imposes three types or levels of obligations on States parties: the obligations to *respect*, *protect *and *fulfil*. In turn, the obligation to *fulfil *contains obligations to *facilitate*, *provide *and *promote*. The obligation to *respect *requires States to refrain from interfering directly or indirectly with the enjoyment of the right to health. The obligation to *protect *requires States to take measures that prevent third parties from interfering with article 12 guarantees. Finally, the obligation to *fulfil *requires States to adopt appropriate legislative, administrative, budgetary, judicial, promotional and other measures towards the full realization of the right to health [25]." States parties are legally obliged to give sufficient recognition to the right to health in the national political and legal systems, preferably by way of legislative implementation, to adopt a national health policy with a detailed plan for realizing the right to health, and the necessary level of investment to enable effective implementation of such policies and plans. These obligations apply to physical and mental health.

## Discussion

### What should be done?

Governments must acknowledge that human rights abuses are common and widespread, initiate practical actions to eliminate systematic abuses and to discharge their international and domestic legal and moral obligations. This will require the implementation of non-discrimination principles, and equality before the law, in areas such as access to education, employment, housing and social security. Access to mental health services is a basic right. Elimination of human rights abuses such as pasung or other degrading or harmful treatment is a minimum requirement. Particular attention must be paid to those who are most vulnerable and most in need of human rights protection. Government commitment to the rights of people with mental illness, and investment to ensure that people with mental illness are able to exercise their rights, is essential.

The provision of basic mental health services relies on sufficient numbers of adequately trained mental health workers from all the necessary disciplines, availability and equitable distribution of appropriate health facilities, and availability of medicines and other essential supplies. This will require substantial new investment by governments at all levels.

Professionals must be more alert to, and active in preventing, human rights violations. As Dharmono has reported [22] it is sometimes mental health and other health professionals who are perpetrators of human rights violations. On those occasions when mental health workers themselves are responsible for violating the rights of patients they should feel the full weight of the law. All mental health professionals must become active in protecting and promoting the human rights of mentally ill persons, and for ensuring that treatment settings such as mental hospitals are not systematically violating the rights of mentally ill persons.

The National Human Rights Commission must lead in identifying and investigating violations of the human rights of persons with mental illness, and in assisting governments to recognise and to act on their legal obligations.

The most important element is a community that is well-informed and sufficiently well organised to ensure that people with mental illness are able to effectively assert their rights and can participate fully in the social and political life of the community. Persons with mental illness and their families must become better organised and more skillful advocates on their own behalf. They should form organisations and associations that can engage in a confident and effective manner in discussions and decision-making about mental health systems and advocate effectively for the rights of their members. Governments at all levels should provide financial and technical support for the establishment and growth of such advocacy organisations and establish mechanisms to work respectfully and collaboratively with them.

### Some positive developments

Despite the record of neglect of the human rights of people with mental illness, there have been some positive recent developments.

In the last several years the National Human Rights Commission has taken an increased interest in health and human rights. In the latter part of 2008 discussions were initiated on the issue of mental health and human rights between the Advocacy and Human Rights Working Group of the National Taskforce on Mental Health System Development in Indonesia and the Human Rights Commission. A workshop on mental health and human rights is planned to occur in 2009. The role of the Commission will be central to the development of the multiple strategies that will need to be pursued to protect the rights of persons with mental illness and will support the development of civil society organisations that pursue this objective. Specific attention will be paid to reporting on the human rights of persons with mental illness in the Commission's annual reports.

Discussions in the National Taskforce on Mental Health System Development in Indonesia have also led to the establishment of the Indonesian Mental Health Association, a body that has in its membership consumers, carers and mental health professionals. A delegation from the Association met in February 2009 with members of the national parliament to advocate on behalf of people with mental illness and their families. The Association, and other civil society organisations, will be crucially important in informing the general community about the situation of people with mental illness in Indonesia and in assisting people with mental llness and their families to effectively assert their basic human rights. Empowerment programs for people with mental illness and their families will be an important element of strategies to protect human rights.

Recently the government of the Province of Aceh announced [20] a program for the elimination from that province of the practice of pasung, an extreme form of human rights abuse. This is a very significant human rights development with wide implications for improvement in the level of protection of the human rights of people with mental illness.

### More than legislation is needed

Specific mental health legislation would clarify the rights of people with mental illness and would provide specific protection of rights [26]. However, even in the absence of such legislation there is sufficient basis in Indonesian law for protection of the rights of people with mental illness. Despite the protections that are available in law, and a human rights infrastructure [3], violation of the rights of people with mental illness remains widespread and largely unnoticed.

People with mental illness and their families do not excercise their rights, through lack of awareness that they have such rights, lack of knowledge about how to effectively assert their rights, and lack of confidence in asserting their rights. Non-governmental organisations do not advocate effectively on behalf of people with mental illness, and mental health professionals and health authorities do not adequately protect the rights of people with mental illness or assist them to effectively assert their rights. The National Human Rights Commission has not, until recently, acted on behalf of people with mental illness.

Health professionals and health authorities are most closely aware of human rights abuses and will need to play an active role in eradicating such abuses and preventing them from occurring in health institutions. This will require a clearer awareness of the rights of people with mental illness and a much more critical approach to clinical and institutional practices. The conditions in many mental hospitals are not acceptable and health professionals should not continue to accept them.

A basic requirement for adequate protection of the rights of people with mental illness is the provision of hospital and community mental health services that meet minimum standards of accessibility, affordability and quality. This is a responsibility of governments at national, provincial and district levels. This responsibility cannot be met without adequate investment in mental health services, and the level of investment in mental health in Indonesia is very substantially below what is required for even minimal service provision. Investment in mental health service provision must be substantially increased to enable basic protections of the human rights of people with mental illness. This is not optional. It is an obligation under international laws that Indonesia has ratified.

## Conclusion

Abuses of the human rights of people with mental illness are widespread in Indonesia, as they are in many other countries. Although there is sufficient basis in Indonesian law for adequate protection of the rights of people with mental illness the provisions of the Indonesian constitution and various health-related and other relevant legislation have not been used to protect the rights of people with mental illness. This situation is similar to other countries in which there is a wide gap between the human rights legal framework and aspirations and the reality for people with illness [27]. Specific mental health legislation is, in itself, not enough. Eradicating human rights abuses and positively protecting the rights of people with mental illness will require multiple strategies to be pursued and multiple players will need to be actively involved. The National Human Rights Commission, civil society organisations, and health professionals and health service provision agencies all have important roles to play. The critically important responsibilities lie with national, provincial and district governments. Chief among these is the responsibility to invest adequately in the provision of mental health services that meet minimum standards. Despite the long record of neglect of the rights of people with mental illness there have been important positive developments. Building on these developments will require long-term sustained action that has the rights of people with mental illness as its constant focus.

## Competing interests

The authors declare that they have no competing interests.

## Authors' contributions

All authors contributed equally to this work, and all have read and approved this manuscript.
